# Determining the Diet of Larvae of Western Rock Lobster (*Panulirus cygnus*) Using High-Throughput DNA Sequencing Techniques

**DOI:** 10.1371/journal.pone.0042757

**Published:** 2012-08-21

**Authors:** Richard O'Rorke, Shane Lavery, Seinen Chow, Haruko Takeyama, Peter Tsai, Lynnath E. Beckley, Peter A. Thompson, Anya M. Waite, Andrew G. Jeffs

**Affiliations:** 1 Leigh Marine Laboratory, University of Auckland, Warkworth, New Zealand; 2 School of Biological Sciences, University of Auckland, Auckland, New Zealand; 3 National Research Institute of Fisheries Science, Yokosuka, Japan; 4 Department of Life Science and Medical Bioscience, Waseda University, Shinjuku-ku, Tokyo, Japan; 5 School of Environmental Science, Murdoch University, Murdoch, Western Australia, Australia; 6 Australian Commonwealth Scientific Industrial and Research Organisation, Hobart, Tasmania, Australia; 7 School of Environmental Systems Engineering and the Oceans Institute, University of Western Australia, Crawley, Western Australia, Australia; University of Connecticut, United States of America

## Abstract

The Western Australian rock lobster fishery has been both a highly productive and sustainable fishery. However, a recent dramatic and unexplained decline in post-larval recruitment threatens this sustainability. Our lack of knowledge of key processes in lobster larval ecology, such as their position in the food web, limits our ability to determine what underpins this decline. The present study uses a high-throughput amplicon sequencing approach on DNA obtained from the hepatopancreas of larvae to discover significant prey items. Two short regions of the 18S rRNA gene were amplified under the presence of lobster specific PNA to prevent lobster amplification and to improve prey amplification. In the resulting sequences either little prey was recovered, indicating that the larval gut was empty, or there was a high number of reads originating from multiple zooplankton taxa. The most abundant reads included colonial Radiolaria, Thaliacea, Actinopterygii, Hydrozoa and Sagittoidea, which supports the hypothesis that the larvae feed on multiple groups of mostly transparent gelatinous zooplankton. This hypothesis has prevailed as it has been tentatively inferred from the physiology of larvae, captive feeding trials and co-occurrence in situ. However, these prey have not been observed in the larval gut as traditional microscopic techniques cannot discern between transparent and gelatinous prey items in the gut. High-throughput amplicon sequencing of gut DNA has enabled us to classify these otherwise undetectable prey. The dominance of the colonial radiolarians among the gut contents is intriguing in that this group has been historically difficult to quantify in the water column, which may explain why they have not been connected to larval diet previously. Our results indicate that a PCR based technique is a very successful approach to identify the most abundant taxa in the natural diet of lobster larvae.

## Introduction

Despite considerable research into the biology of spiny lobsters (Family Palinuridae), the larval phase of their lifecycle remains enigmatic. In particular, the composition of the diet of larvae remains uncharacterised for all species of spiny lobster [1], including the western rock lobster (*Panulirus cygnus*), which is the basis of the second largest commercial spiny lobster fishery in the world [2].

Spiny lobsters have an unusually long planktonic larval phase and *P. cygnus* has an estimated larval duration of 9 to 11 months that is spent in oceanic waters extending from the continental shelf margin to over 1,500 km offshore from Western Australia [3]. After this oceanic phase the larvae, which are known as phyllosomata (singular: phyllosoma), are thought to be carried shoreward by ocean currents and eventually undergo metamorphosis into nektonic post-larvae, or pueruli, which actively migrate back onshore [4]. For *P. cygnus* pueruli there is good evidence that the magnitude of recruitment to the coast of Western Australia is positively correlated with westerly winds [5], La Niña events, and the associated increase in strength of the Leeuwin Current [5]–[7]. However, the underpinning causal details of how these oceanic events impact lobster ecology and recruitment to the coastal benthic stock remain to be fully established.

All evidence indicates that the puerulus is a non-feeding phase that fuels its shoreward migration by metabolising extensive lipid reserves built up during the preceding phyllosoma phase which actively feeds in the pelagic environment [8]–[10]. For example, the puerulus of *P. cygnus* caught closer to shore have markedly lower lipid reserves than those caught further offshore [8], a phenomenon consistent with observations of the pueruli of other species of spiny lobster [11], [12]. Settled *P. cygnus* pueruli also display seasonal fluctuations in lipid reserves indicating environmental effects on available energy that may be due to increased metabolic activity from elevated temperature, having to traverse ocean currents and meso-scale oceanic features, or seasonal changes in pelagic prey availability to phyllosomata [9]. Pueruli that have traversed a greater distance to settle on the coast tend to have more depleted lipid reserves than those that have travelled shorter distances, but this pattern is not consistent [9]. The inconsistency could be due to final stage phyllosomata undergoing metamorphosis without sufficient lipid reserves to migrate successfully to shore. This hypothesis is supported by observations of the red rock lobster, *Jasus edwardsii*, for which 16.5% of nektonic pueruli were estimated to have insufficient lipid to reach settlement sites on the coast [13]. Likewise, estimates based on the biomechanics of swimming by spiny lobster pueruli confirm that the lipid energy reserves are marginal for ensuring settlement success [14]. Puerulus mortality due to exhaustion of lipid reserves is presumably more of a risk in *P. cygnus*, which has significantly smaller larvae and post-larvae and therefore a reduced “capacity to store lipid” [8]. Any hypothesis based on the feeding of phyllosomata and their nutritional status at metamorphosis is contentious and, currently, not experimentally testable because it is neither known what the prey of phyllosomata are, nor what ‘triggers’ metamorphosis [4], [15]. The present study examines the efficacy of a molecular approach to identify the prey so that future studies can assess the impact of oceanographic events on prey abundance and health. This would be timely because a recent collapse in puerulus recruitment to the Western Australia coast has dramatically impacted the fishery [16], which has financial as well as ecological implications. Rock lobsters are Australia's most valuable fishery and Western Rock lobsters contributed over 60% of the catch up to the 2003–2004 financial year, but this contribution has trended down to 50% for 2009–2010 [2]. Following this trend the total export value of rock lobsters has declined from over AUS $600 million for 2003–2004 to under AUS $400 million for 2009–2011 [2].

Determining the diet of spiny lobster larvae has been difficult, because the low densities and patchy distribution of these animals in the open ocean makes them difficult to observe and it is perceived to be prohibitively expensive to conduct blue water field studies [17]. Microscopic analysis of the gut contents of phyllosomata is also a difficult way to determine diet because many potential prey lack hard parts and often have transparent body morphology [1]. Researchers have therefore relied on various methods to infer the diet of phyllosomata of several species. These methods include fatty acid profiling of wild phyllosomata [8], [18], captive feeding trials [19], [20], examining limb, gut and mouthpart physiology [21]–[23], enzyme profiling [24], stable isotopes [25], and the sequencing of DNA from gut contents [26]–[29]. These methods, combined with insights from the artificial culture of phyllosomata, suggest that these larvae may be generalist predators that consume gelatinous zooplankton such as Chaetognatha (arrow worms), Cnidaria, fish larvae, Salpa and soft-bodied arthropods. However, many details are still uncertain, including which species or taxonomic groups are targeted in the wild, and how this changes spatially, temporally and with developmental stage. Of the methods used to date, the DNA-based approaches have proved to be the most powerful to study the diet of phyllosomata because they have the capability to assign taxonomy to digesta obtained from the larval gut with a degree of resolution that other methods are not capable of.

In previous DNA studies PCR has been performed with universal primers on DNA extracted from the gut contents and a small random selection of PCR amplicons were cloned and sequenced using traditional techniques. In the first study of its type, one *Panulirus japonicus* phyllosoma from the Atlantic Ocean and two from the Pacific were analysed and returned amplicons from the phylum Cnidaria and the sub-phylum Urochordata [26]. The same study found DNA of Urochordata and Cnidaria in the gut of one of two slipper lobsters (Scyllaridae) that were also analysed [26]. A subsequent study used the same methods on eleven phyllosomata and detected DNA from teleost fish in three larvae [28]. However, these PCR reactions generated an overwhelming quantity of lobster (host) PCR amplicons and numerous clones had to be screened prior to sequencing. The problem of host derived amplicons swamping the prey amplicons was significantly reduced in a subsequent study, that utilised a lobster specific peptide nucleic acid clamp (PNA-clamp) [29], a method that selectively blocks predator PCR amplification and therefore enriches prey signal. Although such PCR enrichment techniques have been common in medical contexts for over two decades, they are only a recent innovation in ecological studies [30]–[33]. Another improvement was to target a shorter genomic region [29], an approach that is consistent with the consensus approach to DNA diet studies [34], [35]. By using PNA-clamping, ten out of thirty-nine phyllosomata were found to contain DNA from the phyla Cnidaria, Ctenophora, Arthropoda, Vertebrata (Teleostei) and Chaetognatha [29].

Despite the success of these DNA-based dietary studies, their authors have indicated several shortcomings that require additional innovations to enable an efficient DNA-based method to study the diet of phyllosomata. In particular, their results contained a high occurrence of PCR sequences that are unlikely to be credible prey sequences [26], [28], [29]. Such sequences included host sequence variants (either PCR artefacts or pseudogenes), chimeras and also a high percentage of amplicons derived from microscopic eukaryotes with a questionable role in phyllosoma nutrition [28], [29]. Also, the small quantities of DNA obtained from the guts of phyllosomata makes DNA analyses highly susceptible to exogenous DNA contamination. These issues can be circumvented through sequencing a greater number of amplicons, which would allow genuine sequences to be identified amongst sequences that are PCR and laboratory artefacts [36]. Therefore, the present study employs a high-throughput DNA sequencing approach to attempt to overcome these shortcomings, and represents a significant advance for this methodology for a wide range of such dietary studies. Using these methods over 30,000 amplicons were sequenced from the gut contents of eighteen phyllosomata of *P. cygnus* (stage VI and VII) taken from a single water mass 150 km offshore of Western Australia in an effort to establish if these methods can more accurately determine the composition of the diet of phyllosomata.

## Methods

### Ethics Statement

Collection and preparation of the phyllosoma material was conducted under Western Australian Department of Fisheries Research Permit number 1724-2010-38 and Murdoch University animal ethics permit number R2338/10.

### Sampling

Samples were collected from the RV *Southern Surveyor* (CSIRO, Australia) on 7, 13 and 14 July 2010 (refer Table 1 for geographic co-ordinates). Surface waters were sampled at night with a surface net (1 m^2^ opening, 1 mm mesh and cod-end with 355 µm mesh) for around 10 minutes at less than 3.7 km hour^−1^. On recovery of the net the contents of the cod-end were poured into shallow plastic trays. Phyllosomata were immediately sorted out, preserved in 70% EtOH and then stored on board the vessel at −20°C for later analysis in the laboratory.

**Table 1:** Samples.

ID	MID	MID	Date	Stage	Length (mm)	Latitude	Longitude
1	MID1	MID9	7-Jul	VII	14.2	30.72	113.52
2	MID1	MID10	7-Jul	VII	15.5	30.72	113.52
3	MID1	MID11	7-Jul	VII	17	30.72	113.52
4	MID1	MID13	7-Jul	VII	17.1	30.72	113.52
5	MID1	MID14	13-Jul	VII	14.5	30.70	113.83
6	MID2	MID9	13-Jul	VII	16	30.70	113.83
7	MID2	MID10	13-Jul	VI	13.9	30.70	113.83
8	MID2	MID11	14-Jul	VII	17.2	30.21	113.09
9	MID2	MID14	14-Jul	VI	14	30.21	113.09
10	MID3	MID9	14-Jul	VII	14.4	30.21	113.09
11	MID3	MID10	14-Jul	VII	17.2	30.21	113.09
12	MID3	MID11	14-Jul	VI	10.5	30.21	113.09
13	MID3	MID13	14-Jul	VI	14	30.21	113.09
14	MID3	MID14	14-Jul	VII	17.5	30.21	113.09
15	MID4	MID9	14-Jul	VI	12.5	30.21	113.09
16	MID4	MID10	14-Jul	VI	17.8	30.26	113.05
17	MID4	MID11	14-Jul	VI	14	30.26	113.05
18	MID4	MID13	14-Jul	VI	13.5	30.14	113.34
ext neg	MID7	MID9					
ext neg	MID7	MID10					
PCR neg	MID7	MID11					
PCR neg	MID7	MID13					

Stage, length and source loction of phyllosoma larvae used in study as well as duplicate negative (no template) controls for contamination originating from DNA extraction (ext. neg) and PCR (PCR neg). Date refers to when the sample was collected and length refers to the distance from the top of the cephalic shield to the bottom of the abdomen. MIDs refer to Roche's multiplex identifiers that uniquely identify samples in the 454 GS reaction [49].

### DNA extraction

Species identity of phyllosomata was confirmed by sequencing the mitochondrial cytochrome-oxidase I gene (COI). For this, approximately 1 mm of the fifth pereiopod was removed and DNA extracted using the prepGEM™ extraction kit (Zygem, Hamilton, New Zealand) following manufacturer's instructions, but in 20 µL reagent volume. PCR used the LCO-1490 and HCO-2198 primers and PCR protocol of Folmer et al. [37] except 20 µL reactions were used. Phyllosomata were staged under a dissecting microscope according to the developmental key of Braine et al. [38].

In the laboratory, phyllosomata were rinsed with 600 mL of sterile MQ water using wash bottles to remove any loosely adhering surface contaminants. Phyllosomata were then mounted in solidified 2% agar gel so that their cephalic region was exposed. Gut content was syringed out using individual, sterile, disposable 31 gauge hypodermic needles (Ultra-fine II, Becton Dickinson, Australia). These were first flushed with Chargeswitch™ DNA extraction buffer (Invitrogen, Carlsbad, CA), mounted on a micromanipulator and then carefully inserted into the hepatopancreas of the phyllosomata and care was taken to minimise contact with the animal's exterior. DNA extraction was performed with the Chargeswitch Forensic™ DNA extraction kit following the manufacturer's instructions. All plasticware used was sterile and nuclease free. Dissection and DNA extraction were performed in a UV sterilised laminar flow hood following the recommendation of Blankenship and Yayanos [39]. PCR reactions were set up in a separate UV sterilised PCR hood.

### Design of prey enriched PCR

Phyllosomata potentially prey on a range of zooplankton from phylogenetically divergent phyla, which restricts potential loci to those gene regions with highly conserved priming sites. The DNA from the digesta removed from the hepatopancreas was also likely to be degraded into short fragments. Therefore, the hypervariable v7 and v9 regions of the 18S rRNA were targeted (Table 2). These are flanked by highly conserved priming sites and diverse assemblages of meiofauna and microscopic eukaryotes have been amplified for v7 [40], [41] and v9 [42], [43]. Details on two primers used in this study have been published previously [44], [45]. The other two primers target a similar region to that used in previously published studies, but have been moved slightly to enable the priming of more metazoan phyla, in particular Cnidaria, Ctenophora and Chaetognatha: 18S_v7_con is a version of Uni1304F primer of Larsen et al [46] and 18S_v9_con targets a region slightly 5′ (upstream) of the popular NSF1624 primer [47]. Because these universal primers would otherwise amplify lobster DNA, they were used in conjunction with a PNA-clamp to suppress the amplification of lobster DNA.

**Table 2:** Locus Specific Primers.

Name	Target	Sequence	Reference
M13F_EukB	18S v9	TGT AAA ACG ACG GCC AGT TGA TCC TTC TGC AGG TTC ACC TAC	[44]
M13R_18s_v9_Con	18S v9	CAG GAA ACA GCT ATG ACC CCT TTG TAC ACA CCG CCC	This study
M13R 18S_v7_Con	18S v7	CAG GAA ACA GCT ATG ACG CCG TTC TTA GTT GGT GGA	This study
M13F All18SR	18S v7	TGT AAA ACG ACG GCC AGT CAT CTA AGG GCA TCA CAG ACC	[45]
Lobster_PNA_18S_v7_17mer	18S v7	TTG CGA ACG GAC ACC AC-Lys	This study
Lobster_PNA_18S_v9_18mer	18S v9	CGC TCT TGG ATG TTC TAC-Lys	This study

Primers used in first round of PCR. Tagged fusion primers used in second round follow Roche guidelines [49] and are available on request.

Prey enrichment has been performed in other DNA diet studies using DNA blocking primers with 3′ termini modified to prevent polymerisation [30]. However, the 18S rRNA of some potential prey, specifically the arthropods, differs little from that of lobsters and if a modified DNA primer were used then there might be non-specific binding. Therefore, PNA-clamps were used because their low mismatch tolerance would reduce non-specific PCR enrichment [48], thereby building on the use of PNA to ascertain the diet of larval lobsters by Chow et al. [29]. In contrast to them, this study did not use the PNA to competitively exclude the binding of PNA primers to predator template, but rather to bind downstream of the primers and arrest polymerisation. The benefit of using this arrest approach is that the enrichment is not constrained to targeting regions of DNA where priming sites were immediately adjacent to hyper-variable clamping sites, but could independently target regions that were ideal primer and PNA clamping sites, which allowed us to design a very reliable PCR [33]. Therefore, the PNA in this study can be used to prevent and enrich the prey of any phyllosomata from the Palinuridae or Scyllaridae.

### PCR

PCR amplification was undertaken in two rounds following the “universal tailed amplicons sequencing” method outlined in the GS Junior System Guidelines for Amplicon Sequencing [49]. In the first round of PCR the hypervariable v7 and v9 regions of the 18S rRNA gene were targeted in separate reactions (refer Table 2 for primer sequences). PCR products were diluted 1∶50 and 2.5 µl used as template in a subsequent reaction to add adapter A and B sequences, the 454 GS-FLX Titanium sequencing key and multiplex identifier tags (MIDs). Each PCR was carried out in 25 µL volumes using a GeneAmp 9700 thermocycler (Applied Biosystems Foster City, CA, USA). Reactions contained 1× reaction buffer, 2 mM of MgSO_4_ (Invitrogen) 0.4× BSA (NEB), 0.1 mM dNTPs (Roche), 0.1 µM of forward and reverse primers (IDT), 1 µM of PNA-clamp (Panagene) and 1 unit of Hi-fidelity Platinum Taq (Invitrogen). First round PCRs contained 25 ng genomic DNA or were negative (no template) controls for contamination during DNA extraction or contamination. The first round of the v7 region reaction was carried out at 94°C for 2 min, followed by 28 cycles of 94°C for 20 s, 56°C for 20 s, and 68°C for 12 s followed by a final extension step of 68°C for 30 s. The first round of the v9 region reaction differed, following a protocol of 94°C for 2 min, followed by 30 cycles of 94°C for 20 s, 59°C for 20 s, and 68°C for 12 s followed by a final extension step of 68°C for 30 s. Second round PCR reactions used the following protocol: 94°C for 2 min, 6 cycles of 94°C for 20 s, 58°C for 20 s, and 68°C for 12 s, followed by 14 cycles of shuttle PCR of 94°C for 20 s, then 68°C for 30 s and a final extension step of 68°C for 30 s.

PCR amplicons, which contained 454 GS-FLX *Titanium* fusion primers and MID sequences, were separately cleaned using Ampure XP™ beads (Agencourt) following the manufacturer's instructions. Ampure XP™ beads were calibrated according to Roche 454 GS Junior Methods Manual [50] and amplicons over 200 bp were size selected. Amplicons were run on the Agilent Bioanalyzer (Agilent Technologies, Germany GmbH) with DNA 1000™ chips to check the quality and size distribution of amplicons. Amplicons were then diluted, pooled, re-cleaned with Ampure XP™ and triplicate samples were quantified the using Qubit Fluorometer (Invitrogen) and quality control repeated on the Agilent Bioanalyzer. After quality control, the pooled amplicons were diluted to 1×10^9^ molecules µl^−1^ ready for sequencing following the Roche 454 GS Junior workflow.

### Bioinformatics

Basic bioinformatics were performed using custom PERL scripts and a brief summary follows. Amplicon reads were assorted to individual samples based on 454 MIDs and then split into the v7 and v9 18S rRNA loci using the PCR primer sequence. To quality control the sequencing output, any reads that did not perfectly match the MID or primer sequences were discarded. Reads were dereplicated, trimmed and then BLASTed [51] against *P. cygnus*. A script was subsequently used to BLAST reads that did not match *P. cygnus* to the NCBI nucleotide database as at August 2011. The ten top-scoring BLAST hits were then returned, or in the case where the top BLAST hits were unannotated environmental sequences, the top one hundred hits were returned. Due to the nature of the BLAST (multiple good hits for a given query sequence), a method was adopted that summarised the taxonomic classification by checking for consistency amongst the top 10 hits. In the cases where the most closely-related sequence could not be easily determined, it was required that 6 out of the 10 hits belonged to the same taxon otherwise the taxonomic identity was labelled as “No consensus”. This was summarised at each of the seven taxonomic levels (Kingdom, Phylum, Class, Order, Family, Genus and Species). In the cases where there was a single best BLAST hit, then this was used as the final taxonomic classification. The frequency of different organisms was then returned for each of the seven taxonomic levels. This approach, of clustering sequences based on their best taxonomic hits also mitigates the problem of grossly overestimating taxon richness that can result from directly clustering sequences into OTUs [52].

To assess if there was sufficient sequencing coverage to capture prey richness a rarefaction curve was generated for both the v7 and v9 loci. For this, an OTU approach was determined to be preferential because resolving taxonomy to fine levels is not reliant on the completeness of the reference database. OTUs were generated at 93%, 95% and 97% similarity thresholds and curves were calculated with the software Analytic Rarefaction 2.0 [53] that implements the equations of Tipper [54].

## Results

### PCR amplicon sequences from the hepatopancreas of phyllosomata

A total of 100,693 sequencing reads were returned by the 454 GS Junior. Out of these reads 36,800 passed the stringent quality control requirements that both MIDs and primers had no mismatched bases. The average number of reads for each sample was 2041, and of these, the average number for the v7 locus was 752 (n = 18) and 1289 for the v9 (n = 18).

### Taxonomic identity of DNA from samples

Of the quality controlled reads, 8,628 were a close match to lobster, with 3,837 reads coming from the v7 loci and 4,791 reads coming from v9. Although numerous lobster reads occurred in the dataset, none of these reads contained the PNA-clamp sequence. The negative controls for PCR reactions were empty for v9, but v7 yielded a small number of reads from vascular plants (probably pollen) and mammals (probably humans). The negative controls for DNA extractions yielded more DNA in the v7 and v9 loci, these were also from mammals, vascular plants (v9) and fungi. The negative control for the extraction also contained nine unidentified sequences and seven sequences for Bacillariophyta. As none of these taxa are relevant to this study and are most likely sampling or laboratory contaminants, the negative controls were excluded from the study, as were any occurrences of the taxa identified in the negative controls if they occurred in other samples.

**Figure 1:**
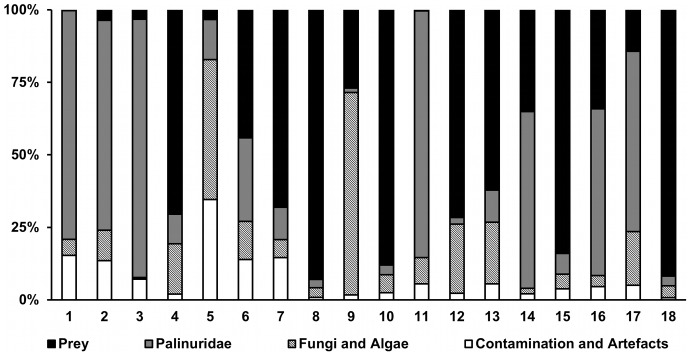
Source of sequence reads among individual larvae. Relative distribution of combined reads for each sample to ascertain the proportion of potential prey reads against other kinds of reads. Samples 1, 2, 3, 5 and 11 had very little potential prey DNA, whereas samples 4, 7, 8, 10, 12, 13, 15 and 17 contained over 50%. Fungal DNA could originate from laboratory contamination, but along with algae, it is just as likely to originate from the gut and is not relevant to the current study. Contamination was either mammalian (probably human) or plant material and sequencing artefacts were mostly very short reads.

Samples 1, 2, 3, 5 and 11 yielded almost no zooplankton sequences and consequently a proportionately higher percentage of contamination, fungus or Palinuridae (i.e. predator) sequence (Fig. 1). This is consistent with there being no metazoan prey tissue in the hepatopancreas of the larvae and the PCR reaction amplifying any available template. These samples were excluded from further analysis. Sample 7 was negative for the v7 loci but yielded ample prey-reads in the v9 region. The sequencing results for sample 7 in the v7 region were similar to those for the v7 negative controls (i.e., contained reads for mammals and fungus); this suggests that there was insufficient template in the initial v7 PCR reaction. Accordingly, sample 7 was only included in subsequent analysis using the v9 region and where the v7 and v9 regions were compared it was excluded.

**Figure 2:**
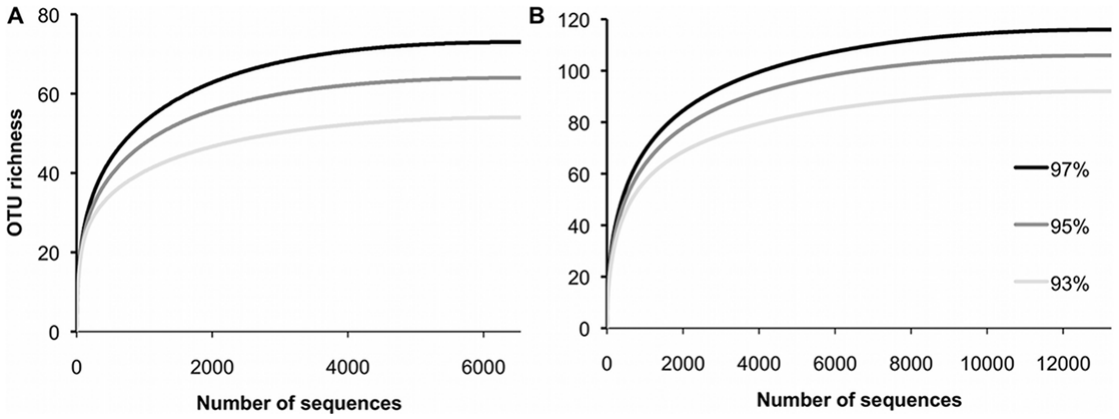
Sampling saturation of (a) v7 and (b) v9 loci. Rarefaction curves representing the number of OTUs detected in pooled samples for the (a) v7 and (b) v9 loci. OTUs are defined at 93%, 95% and 97% respectively and as the percentage threshold increases so does the number of OTUs detected. However, the estimate of OTU richness for each OTU threshold tends toward an asymptote indicating that there is sufficient sequencing coverage to detect most taxa.

**Figure 3:**
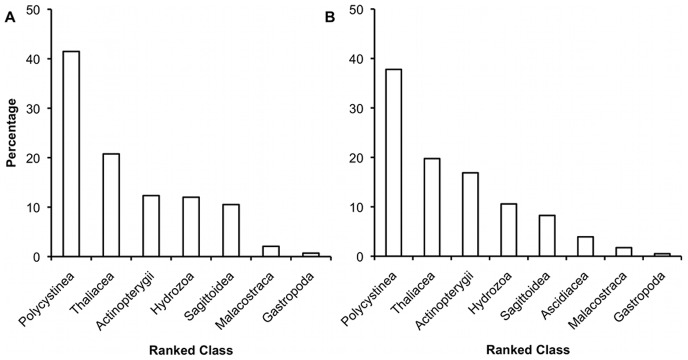
Rank abundance of potential prey reads after standardisation. Rank abundances for the (a) v7 and (b) v9 loci. The x-axis shows the prey classes that constituted more than 0.5% of the sequencing reads. Samples were standardised into ratios of prey per predator prior to combining them into rank abundance.

Once non-prey reads had been determined and removed from samples the average number of prey reads for the v7 locus was 546.7 (n = 12, range: 62–1280) and 1103.1 reads for the v9 locus (n = 12, range: 34–2805). The slopes of the saturation curves rapidly approached asymptotes, which indicates that although there are multiple read types, sufficient sequencing reads were generated to capture major prey items and trends towards capturing full taxon richness (Fig. 2). Rank abundance of DNA reads showed that five taxa were very highly represented in both loci, that less than ten taxa were highly represented overall, and the remainder were represented in very small numbers (Fig. 3). The most abundant reads in both loci, were from the phyla and classes: Radiolaria (Polycistinea), Chordata (Thaliacea), Chordata (Actinopterygii), Cnidaria (Hydrozoa) and Chaetognatha (Sagittoidea), with these five taxa together composing 97.1% of v7 reads and 93.2% of v9 reads. In addition to these top-ranking classes, Malacostraca and Gastropoda contributed about 2% and 0.5% to each locus respectively.

There were minor differences in the results between the v7 and v9 loci with slightly more taxa uncovered using the v9 locus than the v7 locus (Table 3), although, these taxa were generally represented by less than 1% of reads. Ascidiacea (Chordata) occurred exclusively in the v9 locus. Discrepancies are most likely due to PCR amplification biases from primer binding, but these biases are small and do not impact on the detection of significant taxa, or even mildly significant taxa, because there is sufficient coverage to ascertain the range of taxa in a sample, which is evidenced by the species accumulation plot (Fig. 2). The v9 region is more variable than the v7. Therefore, although the same orders of taxa were identified with these loci when a clustering approach is used the v9 assigns reads into more OTUs at similar similarity thresholds (Fig 2). Unfortunately, because of gaps in the reference database for v9 we were unable to resolve any higher order taxonomic assignments with this higher level of information.

**Table 3:** Presence/absence of prey items across samples.

		v7 18S rDNA	v9 18S rDNA
Class	Order	Frequency (n = 12)	
**Malacostraca**	Amphipoda	-	2
	Decapoda	2 (Pandalidae)	2 (Pandalidae)
	Euphausiacea	8 (Euphausiidae)	8 (Euphausiidae)
**Maxillopoda**	Calanoida	1	3
	Calanoida	1 (Metridinidae)	
**Sagittoidea**	Aphragmophora	8 (Sagittidae)	8
**Actinopterygii**	No Consensus	9	7
**Thaliacea**	Doliolida	2 (Doliolidae)	1
	Pyrosomata	2 (Pyrosomatidae)	-
	Salpida	9 (Salpidae)	-
	No Consensus	-	10
**Hydrozoa**	Hydroida	1	1
	No Consensus	2	6
	Siphonophora	7	6
	Trachylina	9	4
**Scyphozoa**	No consensus	-	1
**Tentaculata**	No consensus	-	3
**Holothuroidea**	Apodida	1	2
**Cephalopoda**	Teuthida	3	2
**Gastropoda**	Thecosomata	2	5
	No Consensus	-	2
**Polycystinea**	Spumellaria	12	10

The frequency of prey taxa occurring across the twelve phyllosomata that contained traces of prey in the gut. Prey were identified to the hierarchical level of order and brackets denote the family-level of assignment to a sequence where it could be determined. “No consensus” refers to taxa that have too little database coverage to confidently assign a taxonomic order to the sequences.

## Discussion

### Pyrosequencing

The present study indicates that high throughput sequencing of short 18S rDNA PCR amplicons is a robust solution to the problem experienced in previous DNA diet studies of phyllosomata where the prevalence of non-target amplicons inhibited detection of prey amplicons [26], [28], [29]. High throughput sequencing technologies have had an immense impact on studying bacterial community composition through mass sequencing of the 16S rDNA (e.g. [55]) and have gradually become significant for studies of complex eukaryotic assemblages [56]–[59]. Compared to other high throughput sequencing technology the Roche 454 genome sequencer (454 GS-FLX) enables relatively long read length [60]. While other sequencing technologies are increasing their read length, the substantial lengths achieved with the 454 GS-FLX have made this platform an attractive approach for “bar-coding” based ecological studies including diet studies [31], [61], [62]. The 454 GS has been re-released in a smaller scale “Junior” platform that is affordable for a moderate-sized laboratory (Roche 2011). Using this affordable technology the present study was able to exclude contaminants and chimeras during bioinformatic analyses without having a dramatic impact on the quantity of sequence reads for analysis. This would not be possible with the number of sequence reads afforded in a traditional cloning effort.

By targeting the 18S ribosomal gene the present study overcame the problems that Chow et al. (2010) identified using the ultra-variable ITS1 region, that is, being unable to find sufficiently homologous sequences and the problem of length variation influencing PCR efficiency. However, a disadvantage of targeting short 18S regions is that the reads generally contain only sufficient information to determine their taxonomy to either the order or family level. This disadvantage was a deliberate trade-off considered in our experimental design, but it was more important for our immediate questions that the PCR primers were capable of amplifying all metazoans, which is amply demonstrated by the wide range of taxa revealed from the digesta of the phyllosomata. Few loci besides the 18S rRNA gene have high coverage in public DNA sequence databases and also have primer-binding sites that are conserved across the Cnidaria, Ctenophora, Chaetognatha, Urochordata and Vertebrata. An unexpected benefit of choosing such conserved loci was the discovery of a predominance of DNA from colonial radiolaria, which was otherwise unanticipated and has rarely, if ever, been considered as a potential prey for phyllosomata [1]. Another advantage of the conserved nature of the 18S rRNA is that the PNA-clamps work with all lobster species from the families Palinuridae and Scyllaridae. This is particularly attractive because this protocol and its reagents can be reused to study the structure of the diets of the larvae of other species of lobster, which makes the approach very economical.

There was DNA for multiple taxa in the hepatopancreas of each phyllosoma, with the exception of phyllosoma 7, which returned 339 reads that exclusively matched chaetognatha, indicating Chaetognatha tissue as its sole dietary source. The temptation with community amplicon sequencing is to treat the sequence reads as quantitative or semi-quantitative, and therefore to treat higher frequency operational taxonomic units (OTUs) as having greater biological significance. However, the numbers of each sequence type can be influenced by a multitude of factors beyond prey concentration in the gut. Multi-copy genes such as ribosomal genes vary in copy number across different animals [63]. Read number may also vary due to PCR amplification bias prior to sequencing [64], [65]. Starting with a low quantity of genomic DNA template can also cause stochastic sampling errors to distort amplicon composition [66], [67]. To a limited extent amplification bias was anticipated and controlled by targeting two loci on the same gene. If there were little amplification bias it would be predicted that the same taxa could dominate rank abundance for v7 and v9 (Fig. 3) and that taxa would be ranked in the same order. The concordance between the results for these two loci indicates that PCR bias had little negative impact on determining prey items, and therefore relative abundance of reads at the level of class and order may reflect some relative differences in the consumption of prey species for each phyllosoma. This inference on the relative abundance of reads also tends to be supported by the overall general concordance of these data with the dietary profile among multiple phyllosomata provided by the presence/absence of DNA for the same range of taxa (Table 3).

This study targeted very short reads of around 150 bp (v7) and 180 bp (v9). Although larger DNA fragments are useful for taxonomic assignment, they are in low concentrations in digesta [68] and have proven difficult to amplify in diet studies of phyllosomata [26]. Using very little DNA template and 50 PCR cycles this study generated sufficient amplicons to profile DNA from the hepatopancreas of the larvae, which is an achievement made possible by targeting shorter reads. Results from this study indicate that there are a sufficient number of amplicons generated through PCR, suggesting that fewer PCR cycles could be used in future work, which would be advantageous because it would reduce the accumulation of PCR artefacts. This would not only reduce the number of base miscalls in prey DNA, but also reduce the number of lobster amplicons that have incorporated a replication error in the PNA-clamp region, which enables them to elude enrichment. It might also reduce the discrepancies between the v7 and v9 loci.

The approach presented in this study not only builds on DNA-based approaches to study the diet of phyllosomata, but it is a promising alternative to other methods. The cost of this method was around US $200 per locus per sample. However, because this value includes the initial optimisation of reactions, the purchase of bulk reagents, and due to the ever-decreasing cost of sequencing, it is anticipated that this cost will drop to less than US $100 per locus per sample. This price is more expensive than stable isotope analysis and comparable to the price of lipid analyses that have much less power for taxonomic resolution [8], [18]. Therefore, the better resolution of an enriched meta-genetic approach makes it a valuable tool for trophic analyses of phyllosomata and other pelagic predators.

### Larval diet: taxa from gut DNA

The dataset from this study indicates that gelatinous zooplankton are significant in the trophic ecology of *P. cygnus*. This is entirely consistent with the hypothesised diet items that have been inferred from examining the mouthparts and feeding structures of phyllosomata [1]. Tunicates, fish larvae, siphonophores and chaetognaths have been indicated as prey in the DNA diet studies of *P. japonicus* [26], [28], [29] as well as in experimental feeding of *P. interruptus* [19].

The discovery of DNA from colonial radiolarians in the guts of phyllosomata is a new addition to the groups of organisms associated with the diet of phyllosomata. Colonial radiolarians fit the profile of potential prey for middle to late stage phyllosomata because they are gelatinous zooplankton that cannot easily evade capture [1]. The zooplankton assemblages in East Indian Ocean eddies have typically been assessed using plankton nets, a sampling technique that tends to destroy delicate zooplankton, and probably greatly under-represents colonial radiolarian density [69]. Less destructive sampling techniques such as video plankton recorders (VPR) demonstrate that net tows of identical water bodies under-represent the true concentration of colonial radiolarians by at least an order of magnitude and often by more than several orders [69]. The only video plankton recorder study of East Indian Ocean oceanic waters offshore from the coast of Western Australia found that colonial radiolarians were by far the most dominant member of the zooplankton assemblage [70]. This abundance is not surprising because colonial radiolarians are very well suited to the extreme oligotrophic conditions found in this oceanographic region. The colonial radiolarian DNA identified in this study came from the families Sphaerozoidae and Collosphaeridae, which are monophyletic clades that are exclusively colonial and lack skeletons [71]. The Sphaerozoidae has species whose colonies can reach dimensions of over 2–3 m [72], [73] making them large targets for passive encounter by drifting phyllosomata in the water column, and they could serve as both a refuge and food source for phyllosoma.

### Interpreting multiple reads from hepatopancreas

Recovering amplicons from multiple taxa in each phyllosoma sample was not unprecedented. In their cloning based study Chow et al. [29] detected 0–4 taxa for each phyllosoma and one phyllosoma contained 10 different prey types. Of these reads, thirteen were from Ctenophora, six and four were unidentified homologues and the other seven reads were singletons. The presence of multiple reads could have several explanations; some reads are possibly sampling artefacts, phyllosomata may prey on multiple taxa, some taxa may be from secondary predation, and phyllosomata may engage in coprophagy. These possibilities are discussed in detail below.

Sampling artefacts could result from phyllosomata opportunistically feeding on animals in the cod-end of the zooplankton net or subsequently in sorting trays. Opportunistic net feeding has been reported in ecology studies for other predators [74], however, this seems unlikely as phyllosomata appear to become incapacitated once captured in nets due to the fragility of their feeding appendages. Phyllosomata of slipper lobsters (of the same infra-order as *P. cygnus*) have been observed in the wild to cling to Cnidaria [75], [76], [77]. The Cnidaria could be prey, transport or both. This close association between the animals means that there is a risk that exogenous DNA adhering to the predator's exterior could be detected through PCR and subsequent sequencing. To minimise this risk the exterior of the animals were washed when collected and prior to dissection, also the gut contents were syringed out of the hepatopancreas through a small gauge needle. By taking these measures the overwhelming majority of DNA extracted for this study came from the predator's interior. The presence of multiple taxa inside each phyllosoma may be explained by a recent history of feeding on several different prey items. Examination of phyllosomata in culture conditions has demonstrated that they can be voracious feeders that are capable of rapidly processing prey [20], [78], [79]. The digestive gland structure also includes a series of blind diverticula within which digestion processes may progress relatively slowly in comparison to the rate at which prey may be consumed [78]. Therefore, it is conceivable that the gut contents of phyllosomata are a representation of recent prey feeding history in terms of the residual DNA retained throughout the extent of the digestive gland. Of the eighteen phyllosomata examined, five had no prey DNA signal, possibly due to recent absence of prey encounter, or a temporary halt to feeding which is known to occur when phyllosomata enter a moulting event [80]. Of the remaining thirteen phyllosomata, colonial radiolarian DNA was predominant and was detected in twelve phyllosomata suggesting that it is a commonly consumed prey species. Thaliacea, predominantly Salpa, were the next most abundant class and were present in ten phyllosomata. Actinopterygii, Hydrozoa and Sagittoidea were each detected in nine larvae (Fig 3).

DNA dietary studies can be very sensitive to secondary predation, where the food species that remains present in the digestive tract of the prey is co-amplified with prey DNA [81]. Phyllosomata can rapidly consume their entire prey [1], so it is likely that phyllosomata are also consuming the gut content of their prey. Directly targeting the gut of zooplankton is not an unusual trophic strategy in oligotrophic water [82], as the gut contains a conveniently concentrated and partially digested source of plankton. The gut/pseudogut contents of Urochordata, Cnidaria and Ctenophora are limited only by mouth size and many of the low abundance amplicons detected, such as Bacillariophyta, could well have been the food species of prey. Likewise, colonial radiolarian are known to consume various zooplankton upon making contact with them [83]. Although Chaetognatha are most often described as preying on copepods, this is most likely because most studies on chaetognatha have taken place in eutrophic waters where they are often an abundant and important predator of grazing copepods [84]. However, the Chaetognatha have been found to consume various other prey such as Tinnitids, Appendicularia [85] and Euphausiacea [86]. Chaetognatha also digest only around 80% of their prey [87], which would provide sufficient residual DNA for it to be detected as secondary predation in our study.

Many zooplankton opportunistically consume detritus (marine snow) such as faecal pellets, discarded appendicularian ‘houses’ and zooplankton carcasses [88], [89]. In captive feeding studies *P. cygnus* were observed to frequently capture small pieces of suspended detritus to feed on (pers ob). If phyllosomata consumed detritus it would provide a further explanation for the detection of multiple taxa taken from gut samples. If phyllosomata are coprophagic then the most abundant DNA reads from the hepatopancreas are likely to be from the animal that excreted the particle and the sequences in lower abundance would be traces of that animal's prey. However, because DNA is an excellent source of carbon, phosphate and nitrogen it is unlikely to last long in these conditions, so if the present study detected DNA from faecal pellets then they would have been recently excreted. For example, faecal pellets from tunicates are rapidly colonised and assimilated by microbes and other zooplankton that could rapidly digest residual DNA [90]. If it occurs, then coprophagy is likely to be an opportunistic feeding strategy by phyllosomata and not their primary dietary source because captive feeding studies and the physiology of phyllosomata all demonstrate active predation of zooplankton [20], [78]. However, it is logical that a passive encounter feeder in oligotrophic waters will opportunistically consume marine snow. So despite the strong evidence that phyllosomata are active predators, marine snow and faecal pellets may potentially act as dietary subsidies. Eel larvae, or leptocephali, which like phyllosomata are a common feature of oligotrophic ocean waters, appear to have specialised in feeding on marine snow, especially the faecal pellets of zooplankton and discarded larvacean houses [91], [92].

### Conclusion. Phyllsoma Diet and the Western Australian Fishery

The variety of gelatinous zooplankton that this study detected, and the possibility of a faecal subsidy, support the hypothesis that phyllosomata are generalist, opportunistic feeders of this type of prey. However, while this opportunism might provide a basal level of sustenance, it may be insufficient to deliver excess energy to store as lipids that are needed to fuel the migration of the pueruli to shore. Further work is required to ascertain the optimal food for phyllosomata and whether oceanographic processes impact the density of optimal prey groups and/or the ability of phyllosomata to capture these prey. Attempts to culture phyllosomata have found that feed is a strong determinate of larval viability, and supplementation of feed with mussel gonad has been found to be essential for phyllosomata to advance through multiple instars (Kittaka 1997). More recently, a study of wild-caught mid-late stage *P. cygnus* phyllosomata reared under culture conditions and offered live freshly caught chaetognatha, salpa or krill, showed a clear preference for, and an ability to consume significant numbers of chaetognatha [20]. It was also shown that phyllosomata experimentally fed on chaetognatha over six days tended to accumulate lipid, which is known to be important for energy storage in phyllosomata for subsequent use during the pueruli migration to the coast [8], [13], [20]. The spatial and temporal variability in the availability of these significant prey items could result in phyllosomata in poor nutritional condition, which in turn may contribute to pueruli having insufficient lipid reserves to recruit back to the shallow coastal environment [8], [14]. Together, the results of these studies indicate that if the most nutritionally significant prey items of phyllosomata can be determined, it would allow for the monitoring and modelling of how oceanic dynamics impact on these prey that would improve in the understanding of recruitment and assist in managing the rock lobster fishery.

Without knowing the diet of phyllosomata it is not possible to test the hypothesis that spiny lobster larval recruitment depends on successful feeding of the oceanic phyllosomata to fuel the shoreward migration of non-feeding pueruli. The DNA approach presented in the present study identifies a group of key animals that are clearly important in the trophic ecology of *P. cygnus* phyllosomata. While this approach cannot unequivocally establish that an animal is directly preyed upon, it is highly informative for providing an indication of the potential nutritional importance of prey taxa. For example, the results indicate colonial radiolarians are a predominant constituent of the prey DNA of phyllosomata. Although it cannot be confirmed that phyllosomata feed directly on colonial radiolarians, or via coprophagy or secondary predation, such as through chaetognatha, it remains the first time to our knowledge that colonial radiolarian have been associated with the diet of phyllosomata. Other taxa for which DNA was commonly found in phyllosomata, included salpa, fish larvae, siphonophora and chaetognatha suggesting they are also likely to be significant to the trophic ecology of *P. cygnus*. The high-throughput amplicon sequencing approach presented in the present study enables further discovery of how the trophic connections of *P. cygnus* are spatially and temporally structured and how this relates to the nutritional condition of the phyllosomata.
